# A longitudinal examination of the interpersonal model of binge eating in australian adolescents

**DOI:** 10.1186/2050-2974-3-S1-O58

**Published:** 2015-11-23

**Authors:** Charlotte Lewis, Isabel Krug, Francis Puccio, Matthew Fuller-Tyszkiewicz, Primrose Letcher, Ross King, Craig Olsson

**Affiliations:** 1 University of Melbourne, Melbourne, Australia; 2 Deakin University, Melbourne, Australia

## Objective

To use a large Australian adolescent sample to test the original interpersonal model of binge eating longitudinally and a new version of the model in which bulimic behaviour leads to depression, which in turn leads to interpersonal problems.

## Method

Participants in the current study were part of the Australian Temperament Project. Participants were 1453 (702 females) adolescents and were assessed across five time points: 11-12 years (T1), 13-14 (T2), 15-16 (T3), 17-18 (T4), 19-20 (T5). Interpersonal problems were drawn from parent and self-reported questionnaires at T1 and T5. Data on depression was taken from self-reports at T2 and T4 and data on bulimic behaviour was taken from self-report at T3.

## Results

Structural equation modelling was used to examine both models. The original interpersonal model had acceptable fit, χ2 (df=4, N=1453)= 8.275, p= .082, CFI= .99, RMSEA= .03, SRMR= .01. The new version of the model also had acceptable fit, χ2 (df=1, N=1453)= 10.170, p < .001, CFI= .99, RMSEA=.08, SRMR= 0.02. Depression mediated the relationship between interpersonal problems and bulimic behaviour in both models.

## Conclusions

The results provide longitudinal support for the interpersonal model of binge eating and initial evidence for a new version of the model, offering important insights into the role of interpersonal problems in the development and maintenance of bulimic pathology.

